# An Umbrella Review of E-Cigarettes’ Impact on Oral Microbiota and Biofilm Buildup

**DOI:** 10.3390/pathogens14060578

**Published:** 2025-06-10

**Authors:** Beatriz Panariello, Fabrízio Dias Panariello, Ashminie Misir, Eliane Porto Barboza

**Affiliations:** 1 School of Dental Medicine, Lake Erie College of Osteopathic Medicine, Bradenton, FL 34211, USA; 2 School of Medical Sciences, São Leopoldo Mandic, Campinas 13045-755, Brazil

**Keywords:** e-cigarettes, vaping, electronic nicotine delivery systems, oral biofilms, biofilms, dental plaque, oral microbiota

## Abstract

E-cigarettes, a form of electronic nicotine delivery system (ENDS), have gained significant popularity, particularly among adolescents who often view vaping as a “cool” lifestyle choice. This growing trend has spurred extensive research on the effects of ENDS on both oral and systemic health. By synthesizing data from systematic reviews and meta-analyses, this umbrella review offers a comprehensive evaluation of the impact of e-cigarettes on oral biofilm accumulation and microbiota composition. A systematic search was conducted up to 12 March 2025, across PubMed/MEDLINE, Google Scholar, Cochrane Library, and Scopus for studies published between 2015 and 2025. Ten studies met the eligibility criteria. The quality of the selected papers, as assessed using the AMSTAR 2 tool, ranged from moderate to high. The findings of this review suggest that e-cigarette use may contribute to dysbiosis in the oral microbiota and foster biofilm accumulation, thereby increasing the risk of oral diseases such as periodontitis, peri-implantitis, oral candidiasis, and caries. The findings also highlight the need for further research into the long-term effects of e-cigarette use on oral health. This review is registered with PROSPERO (CRD420251025639).

## 1. Introduction

E-cigarettes are electronic nicotine delivery systems (ENDS) that consist of a power source, a heating element, and a tank or cartridge filled with “e-liquid”, which is a blend of nicotine and flavorings in a glycerol–propylene glycol base [1]. Initially marketed as a safer alternative to traditional smoking, the popularity of e-cigarettes has been rapidly increasing since their introduction in the early 2000s, particularly among adolescents [1,2]. The device’s appeal has been fueled by its variety of flavors, perceived lower health risks, and growing social acceptance [1,2].

According to the Centers for Disease Control and Prevention (CDC) [3], in 2024, e-cigarettes became the most commonly used nicotine product among middle and high school students in the United States, with 1.63 million students currently using them. This includes 410,000 middle school students and 1.21 million high school students. Among those who vape, 87.6% prefer flavored e-cigarettes, and 38.4% report using them on at least 20 of the past 30 days. Since most adolescents begin vaping during this critical developmental stage, it increases the risk of long-term nicotine addiction and associated systemic and oral health issues [1,2,3].

Oral health is intricately linked to a healthy oral microbiota. Disruptions of its microbial balance, referred to as dysbiosis, significantly increase the risk of developing various oral health issues, such as periodontal and peri-implant diseases and caries [4,5,6]. Traditional cigarette smoking has long been recognized as a significant cause of oral dysbiosis [4,7,8,9,10,11]. With the increasing popularity of e-cigarettes, however, it has been noted that these devices can also disrupt the microbial balance in the oral cavity [12,13], and the microbial balance is essential to maintain a healthy oral microbiota [14,15,16,17]. Research has shown that e-cigarette use in both periodontally healthy and diseased individuals is associated with shifts in the composition and function of the oral microbiota, as well as an increase in opportunistic pathogens [1,12,13]. Additionally, ENDS vapors contain environmental toxins, such as reactive aldehydes and carbonyls, which are produced when the liquid components and chemical flavorings are heated [1,13]. These toxins can be absorbed by the body, potentially disrupting the oral microbiota and host cells, thereby compromising oral health [1,4,12,13].

In addition to environmental toxins, commercially available e-liquids have been found to contain fermentable sugars, such as glucose, fructose, and sucrose, added to mask the bitterness and harshness of nicotine, enhance aroma, and stimulate the release of opioids and dopamine [18,19]. Cariogenic bacteria, such as *Streptococcus mutans,* readily utilize these sugars, fostering the overgrowth of acidogenic and aciduric bacterial species, which significantly contribute to the development of dental caries [18]. Beyond caries, the disruption of the microbiota facilitates the proliferation of opportunistic and pathogenic microorganisms, for instance, *Candida albicans*, associated with oral candidiasis, and *Porphyromonas gingivalis,* linked to periodontitis and peri-implantitis [4,5,6,9,16,17,20].

By consolidating findings from systematic reviews and meta-analyses, this umbrella review synthesizes existing evidence to offer a comprehensive understanding of how e-cigarettes influence biofilm accumulation and the composition of the oral microbiota. Additionally, it identifies gaps in the literature to guide future research efforts.

## 2. Materials and Methods

### 2.1. Reporting Format

This umbrella review was conducted following the PRISMA guidelines [21]. We investigated the impact of e-cigarette use on the buildup of oral biofilms, with a focus on alterations in microbial communities, comparing these effects to those associated with traditional smoking and/or non-smoking behaviors. This review is registered with PROSPERO (CRD420251025639).

### 2.2. Population, Exposure, Comparison, Outcome (PECO)

The focused question was proposed using the PECO format: How does e-cigarette use affect the composition of the oral microbiota and oral biofilm buildup compared to non-smokers and traditional cigarette smokers?

Population: E-cigarette users, traditional cigarette smokers, and non-smokers.
Exposure: E-cigarettes or ENDS.
Comparison: Non-smokers vs. traditional cigarette smokers.
Outcome: Biofilm buildup or plaque index and composition of the oral microbiota.

### 2.3. Eligibility Criteria

Inclusion Criteria: Systematic reviews, with or without meta-analyses, that examine the effects of e-cigarette use on the buildup of oral biofilms (e.g., plaque index) and/or the composition of the oral microbiota in e-cigarette smokers, traditional cigarette smokers, and non-smokers. No language restrictions were applied.
Exclusion Criteria: Studies that are not systematic reviews or meta-analyses, studies that do not focus on e-cigarette use, studies that do not address oral health outcomes, commentary papers, and studies involving non-human populations.

### 2.4. Information Sources and Search Strategy

Electronic and manual searches were conducted to identify studies published from 2015 to 2025 that examine oral microbiota and biofilm accumulation in e-cigarette users, up until 12 March 2025. The following electronic databases were searched: PubMed/MEDLINE, Google Scholar, Cochrane Library, and Scopus, with no language restrictions applied. The keywords and Boolean terms used in the search were (e-cigarettes OR vaping OR electronic nicotine delivery systems) AND (oral biofilms OR biofilms OR dental plaque OR oral microbiota OR oral microbiome). The full search strategy for each database is given in Table S1. In addition to the database searches, a hand search was performed by reviewing the reference lists of relevant studies to identify additional articles for inclusion in the umbrella review.

### 2.5. Study Screening and Data Extraction Procedures

The titles, abstracts, and full texts of the search results were independently screened by two reviewers (B.P. and F.D.P.) for inclusion based on the eligibility criteria. Any disagreements were resolved through a blind review process conducted by another reviewer (E.P.B.). Two reviewers (B.P. and A.M.) extracted qualitative data from the studies, while two other reviewers (E.P.B. and F.D.P.) verified the extracted data for accuracy and completeness.

### 2.6. Quality Assessment

The methodological quality of the systematic reviews and meta-analyses included in this umbrella review was evaluated using the AMSTAR 2 tool [22]. Through this tool, the overall confidence in a review’s results is rated as high, moderate, low, or critically low, based on the number and severity of both critical and non-critical weaknesses identified. Critical flaws are major issues that undermine the review’s reliability and may lead to biased or misleading conclusions. These include problems like inadequate study selection, failure to assess bias in individual studies, or neglecting publication bias. In contrast, non-critical flaws, such as failure to justify study exclusions or lack of detailed study descriptions, may affect the thoroughness or transparency of the review but do not fundamentally undermine its findings. A review is considered high quality if it has no more than one non-critical flaw and provides an accurate summary of the available studies. It is rated moderate if it contains multiple non-critical weaknesses but still offers an accurate summary of the included studies. A review is deemed low confidence if it contains one critical flaw that could compromise its accuracy and comprehensiveness. Finally, a review is rated critically low if it has multiple critical flaws, rendering it unreliable for providing an accurate summary of the studies [22].

## 3. Results

### 3.1. Overview of the Screening Process

**Figure 1:**
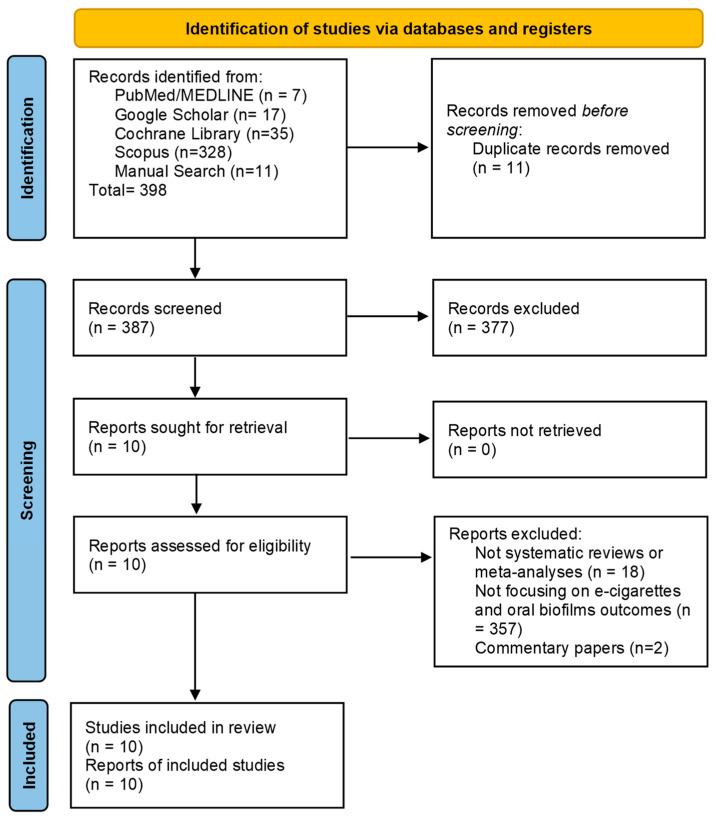
PRISMA flowchart.

We initially retrieved a total of 398 records from electronic databases and manual searches. After removing 11 duplicate records manually, 387 records were screened. Based on the exclusion criteria, 18 papers were excluded because they were not systematic reviews or meta-analyses, 357 studies were excluded for not focusing on e-cigarettes and oral biofilm outcomes, and 2 documents were excluded for being commentaries. A total of 10 full-text articles were retrieved, all of which met the inclusion criteria. This umbrella review synthesizes data from 10 systematic reviews, with or without meta-analysis. The PRISMA [21] flowchart of the study screening process is shown in Figure 1.

### 3.2. Characteristics of the Included Studies

This umbrella review synthesized findings from ten studies that assessed the effects of e-cigarette use on dental plaque accumulation and microbial diversity. The characteristics of the included studies, such as the number of studies included in each systematic review/meta-analysis, population studied, risk of bias and quality assessment methods, sources of funding, outcomes related to plaque accumulation and/or microbial diversity changes in e-cigarette users, and study conclusions, are summarized in Table 1. Heterogeneity was observed across the included studies, with variations in study design, participant characteristics, interventions, and outcome measures. These differences may contribute to the observed variability in findings. While statistical tests for heterogeneity were not conducted, the diverse study characteristics were considered when interpreting the overall results.

Tattar et al. [23] conducted a systematic review of 40 studies examining the periodontal health of e-cigarette users, traditional cigarette smokers, and non-smokers. revealed that e-cigarette use is linked to alterations in the oral microbiome, including shifts in microbial populations that pose oral health risks. This finding aligns with other studies, such as Shabil et al. [24], who reviewed 12 studies to investigate the effects of e-cigarette use on periodontitis. They found that e-cigarette users had a significantly higher plaque index than non-users, which echoes the findings of Tattar et al. in terms of the impact on oral microbiota. Similarly, Vámos et al. [25], in their systematic review of 39 studies, found that e-cigarette users possessing dental implants accumulated more plaque than non-users, though less than traditional cigarette smokers. This pattern is consistent with the results of Zięba et al. [26], who reported an imbalance in oral microbiota among e-cigarette smokers, with periodontal disease severity higher than non-smokers but lower than traditional smokers, suggesting that e-cigarettes contribute to oral health issues, though to a lesser extent than conventional smoking. This is further supported by Youssef et al. [27], whose meta-analysis of four studies showed that e-cigarette users exhibited worse clinical peri-implant parameters in men, including increased plaque index and probing depths. In a similar vein, Camoni et al. [28] found a connection between e-cigarette use and self-reported gingivitis, reinforcing the idea that e-cigarette use negatively impacts oral health. The authors, however, found high heterogeneity in the studies reviewed.

Thiem et al. [29] also noted that cigarette smokers had a higher plaque index compared to e-cigarette users and non-smokers, suggesting a similar but more severe impact of traditional smoking on oral health. Figueredo et al. [30] reviewed studies on the influence of vaping on periodontitis and found that e-cigarette users had consistently higher plaque index scores, supporting the notion that vaping contributes to plaque accumulation. Yang et al. [31] reviewed 98 articles and highlighted that e-cigarette use was associated with increased plaque, deeper probing depths, and more bone loss, underscoring the risk of periodontal disease among e-cigarette users. Finally, Ralho et al. [32] found that although conventional e-cigarette users showed no significant differences in plaque index in six of the eight studies reviewed, they did exhibit a higher prevalence of *Candida* spp. compared to non-smokers. This finding adds another dimension to our understanding of how e-cigarette use can alter the oral microbiome.

Together, the studies included in this umbrella review provide a comprehensive picture of the negative effects of e-cigarette use on oral health, showing that while the impact may vary in severity compared to traditional smoking, it still poses significant risks, particularly in terms of plaque accumulation, changes to the oral microbiome, and increased susceptibility to periodontal diseases.

### 3.3. Quality Assessment Results

The quality of the included studies was assessed using AMSTAR 2 [22]. Nine studies were rated as having moderate quality [23,24,25,26,27,28,29,30,32], while one study was deemed to be of high quality [31]. The sixteen items evaluated for each of the papers included in this umbrella review are summarized in Table S2. A notable characteristic across the nine studies that received a moderate score was the failure to report the sources of funding for the included studies in their systematic reviews. This raises the possibility of potential conflicts of interest that could have influenced the interpretation of their findings. Additionally, the moderate risk of bias in these studies is partially due to failing to search the grey literature, references from the selected papers, or consulting with experts in the field. These shortcomings may result in incomplete findings, as relevant studies might not have been considered. Moreover, all the studies failed to provide a complete list of excluded studies, although they justified the reason for the exclusions. Consequently, while these studies provide valuable insights into the effects of e-cigarettes on biofilm accumulation and microbial diversity, the potential for bias introduced by these methodological weaknesses should be carefully considered when interpreting the results.

## 4. Discussion

The rise in popularity of e-cigarettes, particularly among adolescents who often perceive them as “cool,” has sparked increasing research into their effects on oral health. A key focus of this research has been on biofilm formation and microbial diversity. This umbrella review, which synthesizes findings from ten systematic reviews/meta-analyses, provides essential insights into how e-cigarette use contributes to the accumulation of oral biofilm and alters the composition of the oral microbiota.

Biofilm accumulation, as measured clinically by the plaque index, is a key indicator of oral health. Effective biofilm management requires good oral hygiene, a low-sugar diet, and salivary factors such as proper flow and composition, each of which can be influenced by various systemic factors, including alterations in the immune system, the use of certain medications that can cause an imbalance in the oral microbiota (e.g., antibiotics), and lifestyle habits, such as smoking. When biofilm is not properly managed, the microbial community in the mouth can shift toward pathogenic bacteria, which can lead to oral diseases like caries, periodontal and peri-implant diseases, and oral candidiasis [5,6,33].

A consistent finding across several studies analyzed in the present review was that e-cigarette users exhibited a higher plaque index compared to non-smokers [24,25,27,29,30,31], although this effect was not as pronounced as in traditional cigarette smokers [25,29,31]. Vámos et al. [25] found that non-smokers and e-cigarette users reported similar brushing habits, while cigarette smokers brushed less frequently; still, plaque index in e-cigarette smokers was found to be higher compared to non-smokers. On the other hand, Yang et al. [31] reported that daily e-cigarette use was associated with poorer oral health, including increased tooth loss.

Traditional cigarette smoking has long been associated with an increase in pathogenic bacteria and a decrease in symbiotic bacteria [34,35]. E-cigarette use, while often perceived as a less harmful alternative to traditional smoking, introduces chemicals, such as aldehydes and carbonyls, and additives, such as sugars, that can disturb the oral microbiome in similar ways to traditional cigarettes [1,12,19,36]. Even with good oral hygiene, exposure to chemicals and additives from e-cigarettes can lead to inflammation and increased susceptibility to infections [5,6]. E-cigarette use has been found to change salivary composition and alter immune responses [4,5,6,9]. Studies have reported elevated levels of pro-inflammatory cytokines (e.g., IL-10, IL-12, TNF-α) in e-cigarette users [12], which may contribute to microbiota dysbiosis and an increased risk of periodontal diseases. Furthermore, it was found that e-cigarette users exhibited significantly lower levels of critical antimicrobial proteins in their saliva, such as lysozyme and lactoferrin, compared to non-smokers [12]. This reduction in antimicrobial defense could potentially enhance the susceptibility of e-cigarette users to oral infections.

Tattar et al. [23], Zięba et al. [26], Camoni et al. [28], Yang et al. [31], and Ralho et al. [32] found in their systematic reviews a body of evidence of microbial dysbiosis in e-cigarette users that may predispose individuals to oral infections. For instance, the increased proliferation of *Porphyromonas gingivalis* and *Fusobacterium nucleatum* was observed in ENDS users, suggesting that vaping may promote the growth of pathogens linked to periodontal diseases [36]. These microbial changes were observed to occur rapidly in response to e-cigarette use, with significant alterations in microbial communities within only 24 h of ENDS exposure [13]. Yang et al. [31] observed that e-cigarette users showed a significant worsening of dental caries over six months of use. These findings are consistent with Camoni et al. [28], who highlighted that e-cigarette users exhibited shifts in microbial communities, with an increase in bacteria such as *S. mutans*, a key contributor to dental caries. Ralho et al. [32] found compelling evidence in their systematic review indicating an increased presence of *Candida* species in the oral cavities of e-cigarette users. Their review suggests that e-cigarette use is associated with a higher likelihood of oral colonization by *C. albicans*, an opportunistic pathogen frequently implicated in oral fungal infections such as thrush and denture stomatitis. Camoni et al. [28] also observed an increase in fungal species in the oral microbiota of e-cigarette users. These results reinforce the idea that e-cigarette use has the potential to disrupt the oral microbial balance, fostering an environment conducive to the overgrowth of pathogenic and opportunistic bacteria and fungi.

It is important to note that the quality of the studies included in this review indicated that while the studies generally have no critical flaws, caution is advised due to the potential risk of bias in some of the studies. We noticed that the quality of most of them was reduced to moderate, mostly because of failing to provide a complete list of all the excluded studies. While we acknowledge that providing a list of full references of the excluded studies is important, we understand that it can be challenging, especially when a large number of studies are excluded, so we did not consider this omission a significant flaw. Moreover, we noticed that the statistical analyses across the studies varied, with some reporting significant heterogeneity in their meta-analyses [24,27,28], suggesting that factors such as study design, participant characteristics (e.g., age, sex), geographic location, and different types of e-cigarettes, including variations in flavored vs. non-flavored e-liquids analyzed, may also play a role in the outcomes. This variability underscores the need for more standardized research to better understand the precise impact of e-cigarettes on oral health.

We also noted important discrepancies across the studies included in this review. This is evident in several key aspects, including differences in study populations, methodologies, and outcome measures. For example, the systematic review by Youssef et al. [27] included only male participants with implant-supported prostheses, which limits the generalizability of the findings. Similarly, Vámos et al. [25] only included populations with dental implants, which is a distinct subgroup that could experience more severe oral health consequences from e-cigarette use due to the susceptibility of implants to inflammation and infection [10,16,37]. The focus on dental implants could result in different findings compared to studies that examine natural teeth.

Furthermore, the duration of the studies is a critical factor in assessing the effects of e-cigarettes on oral health. For example, Figueredo et al. [30] examined the effects of e-cigarette consumption on the oral health of individuals who had been vaping for at least one year. While this duration serves as a reasonable baseline for defining long-term use, additional research with longer follow-up periods is essential to fully understand the prolonged effects of e-cigarette use on microbial diversity, biofilm formation, and overall oral health. Future studies should prioritize longitudinal designs to track changes in oral health over time, identify emerging trends, and provide more comprehensive insights into the long-term risks associated with e-cigarette use.

Additionally, more research is needed to explore the specific ingredients in e-cigarette fluids such as nicotine, flavoring agents, and other chemicals to better understand the mechanisms driving microbial shifts and the onset of oral diseases. Gaining insight into how these substances contribute to oral health deterioration will be crucial for developing targeted interventions to mitigate their harmful effects. Furthermore, investigating the impact of e-cigarettes on immune responses in the oral cavity could provide valuable insights into the observed changes in microbial populations and the associated health risks. Given that the immune response plays a critical role in maintaining oral health, any disruptions caused by e-cigarette use may increase vulnerability to infections and other oral health complications.

The findings of this umbrella review highlight that e-cigarette use is associated with increased biofilm buildup and notable shifts in the oral microbiota. E-cigarette users exhibit a higher plaque index compared to non-smokers, suggesting that vaping contributes to biofilm accumulation, though the effect appears less pronounced than in traditional smokers. The microbial shifts observed in e-cigarette users indicate an increased susceptibility to oral infections, such as caries, periodontal and peri-implant diseases, and oral candidiasis, driven by an overgrowth of opportunistic and pathogenic bacteria and fungi. These findings underscore the potential risks e-cigarette use poses to oral health, emphasizing the need for further research to fully understand its long-term impact on oral microbiota composition and biofilm formation. While the findings from this umbrella review provide valuable insights, the observed heterogeneity among studies indicates the need for caution in drawing broad generalizations.

## Figures and Tables

**Table 1:** Summary of the included studies characteristics and main findings.

Authors/Year	Type of Study	No. of Included Studies	Population	Risk of Bias and Quality Assessment Methods	Source of Funding	Outcomes Related to Plaque Accumulation and Microbial Diversity Changes in E-Cigarette Users	Conclusions
Tattar et al., 2025 [23]	Systematic review and meta-analysis	40	E-cigarette smokers, traditional cigarette smokers and non-smokers.	NOS, ROBINS-I, and RoB-2	National Institute of Health and Care Research (NIHR).	Five studies in this review investigated microbiome changes and found that e-cigarette use is associated with alterations in the oral microbiome, with specific microbial shifts tied to oral health risks.	There is evidence that e-cigaretts use has some impact on periodontal parameters compared to non-smokers/former smokers. Tobacco smokers had consistently worst outcomes. Caution is needed due to methodological issues, such as reliance on self-reporting and confounding factors like smoking status and history of the patient.
Shabil et al., 2024 [24]	Systematic review and meta-analysis	12	E-cigarette users and non-users.	NOS	This study received no funding.	There is a significant difference in plaque index between e-cigarette users and non-users, with e-cigarette users exhibiting an increased plaque index.	Further long-term observational studies are needed to better understand the potential risks e-cigarettes pose to periodontal health. The studies showed considerable heterogeneity.
Vámos et al., 2024 [25]	Systematic review and network meta-analysis	39	E-cigarette users, waterpipe users, traditional cigarette smokers, and non-smokers possesing dental implants.	QUIPS	Centre for Translational Medicine, Semmelweis University.	E-cigarette smokers showed significantly higher plaque indexes compared to non-smokers.	Most nicotine-containing product users presented worse clinico-radiographic and immunological peri-implant parameters compared to non-smokers. The analysis revealed no significant heterogeneity across the studies.
Zięba et al., 2024 [26]	Systematic review	65	E-cigarette and traditional cigarette users.	Quality Assessment Tool guidelines issued by the National Heart, Lung and Blood Institute (NHLBI) within the U.S. National Institutes of Health (NIH).	None declared.	E-cigarette use can increase the levels of cariogenic bacteria, such as *Streptococcus mutans* and *Lactobacilli*, in saliva. Nicotine in e-cigarettes is believed to contribute to this by promoting the adhesion of bacteria to the tooth biofilm and stimulating the synthesis of extracellular polysaccharides, which thickens the biofilm. This process may increase the risk of dental caries, as the activity of these bacteria leads to higher levels of lactic acid production.	While some studies show a correlation between e-cigarette use and increased bacterial counts, more research is needed to fully understand the extent of e-cigarettes’ impact on oral health.
Youssef et al., 2023 [27]	Systematic review and meta-analysis	4	Only male e-cigarette users and non-smokers possessing implant-supported prostheses.	Joanna Briggs Institute critical appraisal tool	Did not declare.	E-cigarette users had significantly higher plaque index compared to never smokers.	E-cigarettes may have a negative effect on the clinical, radiographic, and pro-inflammatory profile of dental implants. Further studies, including longitudinal investigations in diverse patient populations, are required to confirm these outcomes.The studies showed moderate but nonsignificant heterogeneity
Camoni et al., 2023 [28]	Systematic review and meta-analysis	84	E- cigarette and heated tobacco smokers or human cells/oral bacteria exposed to ENDS.	ROBIN-I and RoB-2	This study received no funding.	A meta-analyses combining data from 6 studies on self-perceived gingivitis and found an association between e-cigarette use and self-reported gingivitis. They also noted that flavored e-liquids from e-cigarettes are detrimental to the enamel, similar to what is caused by gelatinous sweets or acidic drinks.	E-cigarettes have a potential detrimental effect on periodontal and peri-implant parameters, and laboratory tests confirmed the presence of carcinogenic and inflammatory biomarkers. Flavored e-liquids may be a caries risk factor.
Thiem et al., 2023 [29]	Systematic review and meta-analysis	16	E-cigarette smokers, traditional cigarette smokers and non-smokers.	NOS and GRADE	Open Access funding enabled and organized by Projekt DEAL.	The plaque index was significantly higher in cigarette smokers compared to e-cigarette users and non-smokers in six studies. One study showing no difference between e-cigarette users and non-smokers, and another study showed increased plaque in e-cigarette users compared to non-smokers.	E-cigarette use may be considered a healthier alternative to cigarette smoking in terms of periodontal health; however, harmful effects of e-cigarettes use on periodontal health have also been observed.
Figueredo et al., 2021 [30]	Systematic review	8	Individuals using e-cigarettes for at least one year prior to the study, including both non-smokers and those who did not use any form of tobacco.	Joanna Briggs Institute critical appraisal tool	Did not declare.	Plaque index results are consistently increased across studies with the use of e-cigarettes.	While there is insufficient evidence to fully characterize vaping’s impact on periodontitis, available results suggest a negative effect, highlighting the need for further longitudinal clinical studies.
Yang et al., 2020 [31]	Systematic review	98	E-cigarette users, conventional cigarette smokers and non-smokers using e-ciagaretts for at least one year.	Effective Public Health Practice Project (EPHPP) Quality Assessment Tool	This study received no funding.	E-cigarette users, especially long-term users, were more likely to develop periodontal and gingival disease compared to non-users. They also experienced worse peri-implant parameters than non-users, although better than conventional smokers, along with increased dental issues like cracked teeth, toothache, and worsened dental caries. Daily e-cigarette use was linked to poorer oral health, including tooth loss, and flavored aerosols contributed to decreased enamel hardness and discoloration.	This review suggests that e-cigarette use may reduce negative oral symptoms in conventional smokers but may cause discomfort, lesions, and dental damage in non-smokers. Further research is needed, particularly regarding long-term clinical outcomes, age, and flavored e-cigarette products.
Ralho et al., 2019 [32]	Systematic review	8	Non-smokers, ex-smokers, conventional cigarette smokers, or other types of smokers.	ROBINS-I	This study received no funding.	E-cigarette users did not show significant differences in plaque index compared to non-smokers, except in one study where e-cigarette users exhibited a higher plaque index; however, another study found that e-cigarette users, waterpipe smokers, and cigarette smokers had a higher prevalence of *Candida* spp. compared to non-smokers.	The results suggest that e-cigarettes are less harmful than conventional cigarettes; however, they also indicate that e-cigarette users may be more susceptible to developing alterations in oral biological tissues compared to ex-smokers or non-smokers.

NOS = Newcastle-Ottawa Scale; ROBINS-I = Risk of Bias in Non-randomized Studies of Interventions; RoB = Risk of Bias; QUIPS = Quality In Prognosis Studies; GRADE = Grading of Recommendations Assessment, Development, and Evaluation.

## Data Availability

Data are contained within the article or Supplementary Material.

## Supplementary Materials

The following supporting information can be downloaded at: https://www.mdpi.com/article/10.3390/pathogens14060578/s1, Table S1: Full search strategy for each electronic database.; Table S2: Quality of the included studies evaluated through AMSTAR 2.

## Abbreviations

The following abbreviations are used in this manuscript:

ENDS	Electronic nicotine delivery system
PRISMA	Preferred Reporting Items for Systematic Reviews and Meta-Analyses
AMSTAR	A MeaSurement Tool to Assess Systematic Reviews
