# Supplementary material for: An Umbrella Review of E-Cigarettes’ Impact on Oral Microbiota and Biofilm Buildup
Source: Pathogens. 2025 Jun 10;14(6):578. doi: 10.3390/pathogens14060578 (PMC12196310; doi:10.3390/pathogens14060578)
Supplement: Supplementary file 1 [file pathogens-14-00578-s001.zip › pathogens-3611247-supplementary.pdf]

Table S1: Full search strategy for each electronic database.

**PubMed/MEDLINE:** (E-cigarettes OR vaping OR electronic nicotine delivery systems) AND (oral biofilms OR biofilms OR dental plaque OR oral microbiota OR oral microbiome). Restrict publication date to 10 years and article type to Systematic Reviews. Total= 7 papers
**Google Scholar:** ("e-cigarettes" OR "vaping" OR "electronic nicotine delivery systems") AND ("oral biofilms" OR "biofilms" OR "dental plaque" OR "oral microbiota" OR "oral microbiome") AND "systematic review" -"narrative review" - "review article". Restrict between 2015 and 2025 and only review articles. Total= 17 papers
**Cochrane Library:** "e-cigarettes" OR "vaping" OR "electronic nicotine delivery systems" AND "oral biofilms" OR "biofilms" OR "dental plaque" OR "oral microbiota" OR "oral microbiome". Restrict publication date between January 1st, 2015, to March 12, 2025. Total: 35 papers
**Scopus:** ("e-cigarettes" OR "vaping" OR "electronic nicotine delivery systems") AND ("oral biofilms" OR "biofilms" OR "dental plaque" OR "oral microbiota" OR "oral microbiome") AND "systematic review". Restrict publication date between January 1st, 2015, to March 12, 2025

Table S2. Quality of the included studies evaluated through AMSTAR 2.

**AMSTAR 2 Items**	**Tattar et al., 2025 [23]**	**Shabil et al., 2024 [24]**	**Vámos et al., 2024 [25]**	**Zięba et al., 2024 [26]**	**Youssef et al., 2023 [27]**	**Camoni et al., 2023 [28]**	**Thiem et al., 2023 [29]**	**Figueredo et al., 2021 [30]**	**Yang et al., 2020 [31]**	**Ralho et al., 2019 [32]**
1. Did the research questions and inclusion criteria for the review include the components of PICO?	Yes	Yes	Yes	Yes	Yes	Yes	Yes	Yes	Yes	Yes
2. Did the report of the review contain an explicit statement that the review methods were established prior to the conduct of the review and did the report justify any significant deviations from the protocol?	Yes	Yes	Yes	Partial Yes	Yes	Yes	Yes	Yes	Yes	Yes
3. Did the review authors explain their selection of the study designs for inclusion in the review?	Yes	Yes	Yes	Yes	Yes	Yes	Yes	Yes	Yes	Yes
4. Did the review authors use a comprehensive literature search strategy?	Partial Yes	Partial Yes	Partial Yes	Partial Yes	Partial Yes	Partial Yes	Partial Yes	Partial Yes	Yes	Partial Yes
5. Did the review authors perform study selection in duplicate?	Yes	Yes	Yes	Yes	Yes	Yes	Yes	Yes	Yes	Yes
6. Did the review authors perform data extraction in duplicate?	Yes	Yes	Yes	Yes	Yes	Yes	Yes	Yes	Yes	Yes
7. Did the review authors provide a list of excluded studies and justify the exclusions?	Partial Yes	Partial Yes	Partial Yes	Partial Yes	Partial Yes	Partial Yes	Partial Yes	Partial Yes	Partial Yes	Partial Yes
8. Did the review authors describe the included studies in adequate detail?	Yes	Yes	Yes	Yes	Yes	Yes	Yes	Yes	Yes	Yes

9. Did the review authors use a satisfactory technique for assessing the risk of bias (RoB) in individual studies that were included in the review?	Yes	Yes	Yes	Yes	Yes	Yes	Yes	Yes	Yes	Yes
10. Did the review authors report on the sources of funding for the studies included in the review?	No	No	No	No	No	No	No	No	Yes	No
11. If meta-analysis was performed did the review authors use appropriate methods for statistical combination of results?	Yes	Yes	Yes	No meta-analysis conducted	Yes	Yes	Yes	No meta-analysis conducted	No meta-analysis conducted	No meta-analysis conducted
12. If meta-analysis was performed, did the review authors assess the potential impact of RoB in individual studies on the results of the meta-analysis or other evidence synthesis?	Yes	Yes	Yes	No meta-analysis conducted	Yes	Yes	Yes	No meta-analysis conducted	No meta-analysis conducted	No meta-analysis conducted
13. Did the review authors account for RoB in individual studies when interpreting/ discussing the results of the review?	Yes	Yes	Yes	Yes	Yes	Yes	Yes	Yes	Yes	Yes
14. Did the review authors provide a satisfactory explanation for, and discussion of, any heterogeneity observed in the results of the review?	Yes	Yes	Yes	Yes	Yes	Yes	Yes	Yes	Yes	Yes

15. If they performed quantitative synthesis did the review authors carry out an adequate investigation of publication bias (small study bias) and discuss its likely impact on the results of the review?	Yes	Yes	Yes	No meta-analysis conducted	Yes	Yes	Yes	No meta-analysis conducted	No meta-analysis conducted	No meta-analysis conducted
16. Did the review authors report any potential sources of conflict of interest, including any funding they received for conducting the review?	Yes	Yes	Yes	Yes	Yes	Yes	Yes	Yes	Yes	Yes
**QUALITY LEVEL**	**MODERATE**	**MODERATE**	**MODERATE**	**MODERATE**	**MODERATE**	**MODERATE**	**MODERATE**	**MODERATE**	**HIGH**	**MODERATE**
